# Fibrinolytic Activity of Cultured Cells Derived During Ethylnitrosourea-induced Carcinogenesis of Rat Brain

**DOI:** 10.1038/bjc.1978.63

**Published:** 1978-03

**Authors:** T. A. Hince, J. P. Roscoe

## Abstract

**Images:**


*Br. J. Cancer* (1978) **37**, 424

### FIBRINOLYTIC ACTIVITY OF CULTURED CELLS DERIVED DURING ETHYLNITROSOUREA-INDUCED CARCINOGENESIS OF RAT BRAIN

T. A. HINCE AND J. P. ROSCOE

*From the Department of Cell Pathology, School of Pathology, Middlesex Hospital Medical School, Riding House Street, London, W1P 7LD*

Received 3 October 1977 Accepted 21 December 1977

**Summary.—Using a fibrin-agarose-overlay technique, high levels of plasminogen-dependent fibrinolytic activity have been demonstrated in cell lines derived from an ethylnitrosourea-induced glioma of the rat brain. Cell lines derived from normal adult rat brain showed only low levels of activity. The degree of lysis produced by a cell line was dependent on the average number of cells per colony, and a different pattern of response was observed for tumour and normal cell lines. A good positive correlation existed between the level of fibrinolytic activity, growth in agar and tumourigenicity of a cell line. Fibrinolytic activity was associated with cell lines derived at various times in the latent period, before the appearance of a visible tumour. Many cell lines derived from rat brains at 57–60 (E7), 90–91 (E8) and 111–112 (E6) days after transplacental exposure to ethylnitrosourea showed fibrinolytic activity, and in the latter group the close association with growth in agar and tumourigenicity was also demonstrated. Results from cell lines derived in the E7 and E8 experiments indicated that the possession of fibrinolytic activity preceded the ability of cells to form colonies in agar.**

IN RECENT years, much evidence has accumulated to support the hypothesis that the increased production of plasminogen activators is a characteristic of the transformed state of a cell. Reports have indicated increases in the level of plasminogen-dependent fibrinolytic activity after transformation of early-passage cultures by both viruses and chemical carcinogens (Unkeless *et al.*, 1973; Ossowski *et al.*, 1973; Jones *et al.*, 1976; Pearlstein *et al.*, 1976), in cell lines derived from experimentally produced tumours in animals (Laug, Jones and Benedict, 1975; Pearlstein *et al.*, 1976) and in the cells of, or cell lines derived from, human neoplasms (Laug *et al.*, 1975; Nagy, Ban and Brdar, 1977).

To our knowledge, all previous investigations of the fibrinolytic activity associated with experimentally induced tumours have been carried out on cell lines derived from the gross tumour. However, the period between the initial administration of the carcinogen and the appearance of the visible tumour, the latent period, is often long and contains many changes necessary for the development of the malignant cell. In an attempt to understand these processes, an experimental system in which cell populations may be examined at intervals after the initial carcinogenic dose has many advantages. A system which allows such an approach is the induction of rat brain tumours by ethylnitrosourea (ENU) which, when administered to pregnant rats after the 12th day of gestation, results in nearly all the offspring developing tumours of the nervous system. A large proportion of these (≃60%) are macro- and micro-gliomas of the brain (Druckrey, Ivankovic and Preussmann, 1966; Wechsler, *et al.*, 1969).

Using a sequential *in vivo/in vitro* cell culturing approach (Roscoe and Claisse, 1976) we have examined the properties of cell lines derived from the foetuses or young rats at periods between 2 and 145 days after the administration of ENU to the pregnant mother. The average latent period for the induction of gliomas at the dose used was 246 days. Tumour-like cells with the ability to grow in agar and form tumours on re-injection were observed in cell lines derived at 111–112 days and 138–145 days after carcinogen treatment (Roscoe and Claisse, 1976; 1977). Prolonged culturing of cell lines derived as early as 2 days post-exposure resulted in the appearance of transformed cells, indicating a very early potential for tumourigenicity in rat brain cells exposed transplacentally to ENU (Roscoe and Claisse, 1976).

In this paper we wish to report on the fibrinolytic activity of cell lines derived from an ENU-induced glioma, as well as cell lines derived during the latent period. The possible role and timing of plasminogen-activator production in the progression of transformed cells will be discussed. A preliminary report of this work has already appeared in an abstract (Hince and Roscoe, 1977).

### MATERIALS AND METHODS

*Cell cultures.*—The 2 cloned tumour lines, A15A5, A15A10, were cloned at the 27th transfer of the parent culture derived from an ENU-induced glioma of the rat brain (Lantos, Roscoe and Skidmore, 1976). The 2 non-tumour cell lines, ARBO C9 and ARBO C11, were cloned from a parent culture of normal adult rat brain at the 47th transfer, by Dr C. J. Skidmore in this laboratory. For the cloned cell lines the transfer numbers stated are subsequent to their cloning.

The remaining cell lines, termed 'intermediate stage cell lines' were derived from the cerebra of rats at various times after their transplacental exposure to the carcinogen ENU (40mg/kg) or buffer (Table I). Experimental procedures for the administration of the carcinogen, derivation and maintenance of the cell lines have been previously described in detail (Roscoe and Claisse, 1976). All cultures were maintained in Dulbecco's modification of Eagle's medium (DMEM) containing 15% foetal calf serum (FCS) and sub-cultured when required, usually at weekly intervals.

*Fibrinolysis assay.*—The assay chosen for the determination of fibrinolysis in cultured rat brain cells was based on the fibrin-overlay method of Jones *et al.* (1975). For the study of fibrinolytic activity by individual cell colonies, 60 mm tissue-culture dishes were seeded with 5 ml of a cell suspension (in DMEM +15% FCS) that would result in the growth of 30–60 colonies per dish. After the required period of growth (2–10 days) and before the assay, the growth medium was removed and the colonies washed twice with a phosphate-buffered saline (pH 7·2) solution to remove traces of serum. The 2 solutions (A and B) required for the assay were maintained at 45°C in separate tubes, and mixed in the culture dish to form the agarose-fibrin overlay. Solution A contained 15 μg human plasminogen (Kabivitrum Ltd.) and 3000 μg human fibrinogen (Kabivitrum Ltd.) in 1·5 ml DMEM, and was added to the culture dish first. To this was added Solution B containing 1·8% agarose (Indubiose A.37, Uniscience Ltd) and 10 units bovine thrombin (Parke-Davis Ltd) in 1·5 ml DMEM, and the dish gently rocked to mix the 2 solutions and form the stable agarose-fibrin overlay covering the cell colonies. When required, 15% FCS ε-aminocaproic (1500 μg) and soybean trypsin inhibitor (600 μg) were added to the overlay Solution A. Culture dishes were incubated at 37°C for 18 h, after which time zones of lysis in the agarose-fibrin overlay could be clearly seen by dark ground illumination.

TABLE I.—*Nomenclature of Intermediate-stage Cell Lines*

| Times at which cultures initiated (days since exposure) | Group expt. no. | Cultures from animals exposed to ENU | Buffer |
|---|---|---|---|
| 57–60 | E7 | 43B; 45A, C | |
| 90–91 | E8 | 43D; 45E, F | 47B |
| 111–112 | E6 | 38D, F, G; 40D | 41C, D |

Only lysis zones larger than 2 mm diameter were counted as positive.

Fibrin-agarose gels used in well-diffusion assays for either activating or lytic activity were prepared in a similar manner. For the destruction of contaminating plasminogen the fibrin gels were heated to 80°C for 30 min (Schultz, Wu and Yunis, 1975) and the presence of plasminogen in test solutions demonstrated by the addition of the activating enzyme streptokinase and observing lysis.

The preparation of "harvest fluids", serum-free medium which had been in contact with a sub-confluent monolayer of cells for 18 h, was as generally described by Unkeless *et al.* (1973).

*Determination of cell number and type.*—Concomitant with the assays, duplicate dishes were stained with Leishman's stain and both total colony counts and the number of cells per colony determined by microscopical examination. Where colonies were too large for accurate visual counting, duplicate dishes were trypsinized and the cell number obtained by haemocytometer counts (a method previously shown to give similar results to direct counting when applied to replicate dishes).

To visualize cellular morphology in an area of lysis and allow photomicroscopy of the cells, the differential staining of the fibrin by Coomassie blue and of the cells by Giemsa's stain, as described by Strickland, Reich and Sherman (1976) was used.

*Determination of growth in agar and tumourigenicity.*—The ability of cells to form colonies in soft agar was determined by plating up to $10^4$ cells in 0·3% agar and examining at intervals for macroscopic colony formation. Tumourigenicity tests were performed by injecting 1–2 × $10^6$ cells s.c. into newborn (< 5 day old) syngeneic animals. Further details of these 2 assays have been described previously (Roscoe and Claisse, 1976).

FIG. 1.—Demonstration of fibrinolytic activity of tumour cell line A15A10. After 7 days' growth, colonies (av. 139 cells) were assayed using fibrin-agarose overlay. The plate was incubated at 37°C for 18 h and photographed using dark-ground illumination. Scale marker = 1·0 cm.

### RESULTS

The plasminogen-dependent fibrinolytic activity of cloned cell lines derived from normal adult rat brains and from an ENU-induced glioma has been determined. In addition, cell lines derived from the brains of rats at various times after their transplacental exposure to the carcinogen, but before the appearance of a visible tumour, have been tested for this activity using a fibrin-agarose-overlay assay. All cell lines gave some distinct zones of lysis around plasminogen-activator-producing colonies (Fig. 1). The large zones of lysis produced by a relatively small number of tumourigenic cells were indicated by staining (Fig. 2).

### *Conditions of assay*

In agreement with previous results (Unkeless *et al.*, 1973; Jones *et al.*, 1975) it was found that assays performed in the presence of foetal calf serum gave only low levels of lysis (Table II). When added to plasminogen-containing overlays, FCS reduced the number of lysis zones (Table II) confirming the presence in this serum of inhibitors of plasminogen activation

FIG. 2.—Photomicrograph of single A15A10 colony with associated fibrinolytic zone. The assay was performed after 6 days' growth (av. colony = 64 cells). The plate was incubated at 37°C for 18 h, stained (see Materials and Methods) and photomicrographs of single colonies taken using Wild RG2 red-contrast filter. ×90.

(Unkeless *et al.*, 1973). For this reason, all subsequent assays were carried out in serum-free medium containing added plasminogen (5 μg/ml).

The omission of plasminogen from the assay greatly reduced, but did not abolish, lysis (Table II) which indicated either a plasminogen-independent fibrinolytic activity (Wu *et al.*, 1975) or contaminating plasminogen from another assay component. The plasminogen content of thrombin and fibrinogen was determined by its streptokinase activation, using a well-diffusion assay in plasminogen-free fibrin gels. The level of contaminating plasminogen determined in the thrombin (13% complete assay mixture) could account for the degree of lysis observed in the absence of added plasminogen (Table II). Fibrinolytic activity was inhibited by ε-aminocaproic acid (3·8 mM), at this concentration an inhibitor of plasminogen activation (Alkjaersig, Fletcher and Sherry, 1959) and by soybean trypsin inhibitor.

No fibrinolytic activity could be detected in harvested fluids prepared from a sub-confluent culture of the tumour line A15A5 when these were tested in plasminogen-free fibrin gels.

*Fibrinolytic activity of cloned tumour and normal cell lines*

Table III shows the fibrinolytic activities of two cloned glioma-derived cell lines, A15A5, A15A10 and 2 normal adult rat brain cell lines, ARBO C9, ARBO C11. Lysis zones were produced by all cell lines, although a greater proportion of the tumour-cell colonies showed fibrinolytic activity than the controls (Table III). It was established that the average number of cells per colony greatly affected the number of lysis zones observed per plate.

TABLE II.—*Fibrinolytic Activity of Tumour Cell Line A15A5 under Various Assay Conditions*

| Additions to overlay* | Cells/colony | Colonies showing lysis after 18h incubation: Number/total | % |
|---|---|---|---|
| None | 40 | 20/170 | 12 |
| Plasminogen (15 μg) | 40 | 123/170 | 72 |
| Foetal calf serum (15%) | 34 | 22/213 | 10 |
| Plasminogen+FCS (15%) | 34 | 16/213 | 8 |
| ε-aminocaproic acid+plasminogen | 43 | 0/70 | 0 |
| Soybean trypsin inhibitor+plasminogen | 43 | 0/70 | 0 |

* Assay overlay contained 3000 μg fibrinogen, 10 units thrombin, 0·9% agarose and additions as indicated in table to give final volume, in DMEM, of 3·0 ml.

FIG. 3.—Fibrinolytic activities (% total colonies showing lysis) of cloned cell lines compared on basis of average number of cells per colony at time of assay. ENU-induced glioma-derived cell lines: A15A5, (■); A15A10, (□). Normal adult rat brain cell lines: ARBO C9, (●); ARBO C11, (○). ARBO C11 was further tested at 185 cells/colony and showed activity of 31%, ⓐ

The relationship between fibrinolytic activity and colony size is shown in Fig. 3, where a different pattern of response can be seen in tumour and normal cell lines. This indicated the necessity for comparisons of fibrinolytic activity between cell lines to be made at similar colony sizes; where possible these were 20–50 cells/colony.

When the level of fibrinolytic activity in the tumour and control clones was compared with their abilities to grow in soft-agar or form tumours, there was a good agreement between the production of high levels of plasminogen activator and the possession of the other characteristics of transformed cells (Table III).

### *Fibrinolytic activity of cell lines derived 111–112 days after exposure (E6)*

Six cell lines derived from rat brains 111–112 days after transplacental exposure to carcinogen or buffer were examined for fibrinolytic activity. Cell lines 41C and 41D, prepared from the brains of animals exposed to buffer, both showed low levels of lysis (Table IV). Cell line 38D has been shown to contain a particular cell type, pyramidal basal-layer cell, characterstic of tumour cultures (Roscoe and Claisse, 1977). Cells from 38D formed colones in both soft agar and tumours after only a short latent period (Roscoe and Claisse, in preparation) and have here been shown to have a high fibrinolytic activity (Table IV). In comparison, cell line 40D, which contained a different basal-layer cell type, had a lower plating efficiency in soft agar, a longer latent period for tumour induction (Roscoe and Claisse, in preparation) and a lower fibrinolytic activity than 38D (Table IV). However, subsequent passaging of cell line 40D resulted in the rapid

TABLE III.—*Fibrinolytic activity, Tumourigenicity and Growth in Agar of Cloned Rat Brain Cell Lines*

| | Fibrinolytic activity | | | Tumourigenicity | | | Growth in agar | |
|---|---|---|---|---|---|---|---|---|
| Cell line | Transfer no. | Cells/ colony | % colonies giving lysis | Transfer no. | Incidence | Average latency (days) | Transfer no. | Plating efficiency (%) |
| *From an ENU-induced glioma* | | | | | | | | |
| A15A5 | 13 | 19 | 47 | 6 | 4/4 | 33 | 15 | 29·6 |
| A15A10 | 47 | 29 | 37 | 33 | 4/4 | 42 | 30 | 39·5 |
| *From normal adult rat brain* | | | | | | | | |
| C9 | 96 | 22 | 5 | 70 | 0/6 | 0 | 71 | 0·0 |
| C11 | 15 | 37 | 15 | 15 | 0/5 | 0 | 24 | 0·0 |

TABLE IV.—*Fibrinolytic Activity and Ability to Grow in Agar of E6 (111–112 day) Cell Lines*

| Cell line | Transfer no. | Cells/colony | Colonies showing lysis after 18h incubation: Number/total | % | Ability to grow in agar |
|---|---|---|---|---|---|
| 41C | 30 | 37 | 26/232 | 11 | — |
| 41D | 22 | 26 | 1/65 | 2 | — |
| 38D | 23 | 17 | 121/138 | 88 | ++ |
| 38F | 41 | 55 | 16/100 | 16 | — |
| 40D | 33 | 35 | 43/161 | 27 | + |
| 38G* | 59 | 35 | 101/229 | 44 | + |

* Only tested at late transfer after agar-growth noted.

TABLE V.—*Fibrinolytic Activity, Growth in Agar and Tumourigenicity of Cell Lines 40D and 38F at Various Transfer Numbers*

| | Fibrinolytic activity | | | Growth in agar | | Tumourigenicity | | |
|---|---|---|---|---|---|---|---|---|
| Cell line | Transfer no. | Cells/colony | % colonies showing lysis | Transfer no. | Plating efficiency (%) | Transfer no. | Incidence | Average latency (days) |
| 40D | 33 | 35 | 27 | 29 | 0·154 | 9 | 4/4 | 87·7 |
| | 41 | 35 | 74 | 40 | 0·793 | 37 | 8/8 | 25·0 |
| 38F | 41 | 55 | 16 | 43 | 0·000 | 15 | 0/3 | * |
| | 62 | 53 | 44 | 50 | 0·600 | 48 | 5/5 | 50·5 |

* No tumours at 465 days.

change of basal-layer cell type to that obtained in 38D. When retested for the ability to grow in agar and form tumours, it was found that cell line 40D had become considerably more tumourigenic and that this change was accompanied by an increase in fibrinolytic activity (Table V).

Cell line 38F, in which basal layer cells were lost from the culture after early passages, showed low fibrinolytic activity and no growth in agar, when tested around the 40th transfer (Table V). However, when tested at later passages, it was found that the culture had obtained the characteristics of a tumour culture and showed high fibrinolytic activity (Table V). Cell line 38G was also tested at a late passage and found to have both a high fibrinolytic activity and the ability to form colonies in agar (Table IV). Tests on the control cell line 41C at the 67th transfer still showed only low levels of fibrinolytic activity (13% at 50 cells/colony).

*Fibrinolytic activity of cell lines derived 90–91 days (E8) and 57–60 days (E7) after exposure*

The fibrinolytic activity of the E8 culture derived from the brain of a buffer-exposed animal (47B) was low (Table VI) and the cell line did not grow in agar. The E8 cell lines derived from the brains of animals exposed to ENU showed variations in fibrinolytic activity (Table VI). None of the cultures formed definite colonies in agar at passages similar to those of the fibrinolysis assays. However, in cell line 45F the cells remained alive in agar for much longer periods than observed with the control culture, 47B.

All cell lines tested in the E7 experimental group were derived from the brains of animals exposed to ENU, and in common with E8 cultures, showed variations in fibrinolytic activity (Table VI). At the time of fibrinolysis assays no E7 cell lines grew in agar, although after a

TABLE VI.—*Fibrinolytic Activities of E7 (57–60 day) and E8 (90–91 day) Cell Lines*

| | Cell line | Transfer no. | Cells/colony | Colonies showing lysis after 18h incubatiion: Number/total | % |
|---|---|---|---|---|---|
| E7 | | | | | |
| | 43B | 22 | 35 | 61/147 | 41 |
| | 45A | 26 | 27 | 72/135 | 53 |
| | 45C | 28 | 42 | 31/189 | 16 |
| E8 | | | | | |
| | 47B | 30 | 36 | 17/185 | 9 |
| | 43D | 45 | 22 | 45/251 | 18 |
| | 45E | 31 | 49 | 34/123 | 28 |
| | 45F | 24 | 38 | 49/110 | 45 |

further 12 passages cell line 45A acquired this ability.

### DISCUSSION

We have shown that high levels of fibrinolytic activity were associated with cell lines derived from an ENU-induced glioma of the rat brain. These cell lines, A15A5 and A15A10 have been shown to possess an enzyme inducibility associated with glial cells (Claisse and Roscoe, 1976) and have been characterized as fibrillary astrocytes (Lantos *et al.*, 1976). In comparison to tumour cells, normal brain cells showed only low levels of fibrinolytic activity when compared at similar colony sizes. A positive correlation has been indicated between the level of fibrinolytic activity in cell lines and their possession of other characteristics of transformation. The demonstration of fibrinolytic activity in cell lines derived as early as the first quarter of the latent period raised the question of the possible role of plasminogen-activator production in the progression of maglignantly transformed cells; this will be discussed below.

The use of tumour (A15A5, A15A10) and normal adult rat brain (ARBO C9, ARBO C11) cloned cell lines indicated the importance of determining cell-colony size when comparing the fibrinolytic activities of different cell lines by the fibrin-agarose-overlay method. It appeared important that determinations be made at various colony sizes so that a pattern of response was obtained. This was carried out for all cell lines, although only the graph for the tumour and control clones is shown (Fig. 3). Such graphs showed that comparisons between cell lines should be made at 20–50 cells/colony, where it was found that fibrinolytic activities $<20\%$ were generally associated with control cultures or cells showing no other characteristics of transformation. It is of particular interest that the control cell line, ARBO C9, has maintained a "normal" phenotype after $\sim 4$ years in culture (126 passages since cloning).

Previous reports on fibrinolytic activities have indicated 2 distinct types: one plasminogen-dependent, the other a plasminogen-independent fibrinolysin (Wu *et al.*, 1975). In this study the inhibition of activity by $\epsilon$-aminocaproic acid, its variation with different sera (unpublished observation) and its drastic reduction in the absence of added plasminogen, indicated a plasminogen-dependent activity. This was confirmed by our inability to demonstrate any lytic activity in fluids harvested from a tumour culture, when tested in plasminogen-free fibrin gels, as was observed by Wu *et al.* (1975) with a direct-acting fibrinolytic activity secreted by rat breast carcinoma cells. Our results support the finding of Rifkin and Pollack (1977) that a cell line from an ENU-induced neural tumour had a very high ratio of cell-associated fibrinolytic activity to secreted activity, when compared to other transformed cell lines. At present the basis for the plasminogen-dependent fibrinolytic activity of transformed cells is thought to be as follows. The proenzyme plasminogen is activated by cell-membrane-associated plasminogen activators, which have been characterized as arginine-specific serine proteases with molecular characteristics very similar to the "normal" activatory urokinase, to give an active proteolytic enzyme (Christman and Acs, 1974; Quigley, Ossowski and Reich, 1974; Unkeless *et al.*, 1974; Åstedt and Holmberg, 1976; Wu, Arimura and Yunis, 1977).

Many cell lines derived from the brains of ENU-exposed animals during the latent period showed higher fibrinolytic activities than those derived from animals exposed to buffer. In the E6 experimental group (111–112 days after exposure) the presence of high fibrinolytic activity could be correlated with the ability of cells to form colonies in soft agar. The close association between these 2 markers for transformation was emphasised by results obtained with cell lines 38F and 40D. In each case further passaging of the cell lines resulted in both an increase in fibrinolytic activity and a higher plating efficiency in soft agar (Table V). However, since tests on the 2 cell lines were not carried out at every transfer, it was not possible to determine a definite temporal sequence. Results obtained by Pollack *et al.* (1974) clearly indicated a very close association between growth in methyl cellulose and fibrinolytic activity. In their test system, the highest plating efficiency in methyl cellulose was observed with a high plasminogen-activator-producing cell line tested in a high plasminogen-content serum (Pollack *et al.*, 1975). In general, the reports of other studies have also indicated the close relationship between growth in agar and fibrinolytic activity (Laug *et al.*, 1975; Jones *et al.*, 1976; Rifkin and Pollack, 1977). However, there have been instances, apart from reports of the high fibrinolytic activity of normal kidney and lung cells (Laug *et al.*, 1975; Bernik and Kwaan, 1969) where carcinogen-transformed cell lines able to grow in agar have not shown high fibrinolytic activity (Jones *et al.*, 1976). The separation of fibrinolytic activity from growth in agar has been reported by Leavitt *et al.* (1977) where a mutation causing the loss of hypoxanthine phosphoribosyltransferase activity resulted in the inability of transformed Syrian hamster cells to grow in agar, whilst they retained high fibrinolytic activity.

The question of the causal or coincidental nature of the relationship between fibrinolytic activity and growth in agar is raised by this and other studies (Pollack *et al.*, 1974, 1975; Pollack and Rifkin, 1975). This investigation has shown that cell lines acquired the ability to grow in agar several passages after fibrinolytic activity was first demonstrated (45A of E7 experimental group). It has also been noted with cell line 45F and cell lines derived very soon after exposure to ENU (clones BE10–7, BE10–13, Roscoe and Claisse, 1976) that cells possessing fibrinolytic activity had the ability to survive in agar without forming colonies (unpublished observations). However, with further transfers, clones BE10–7 and BE10–13 formed colonies in agar and became tumourigenic (Roscoe and Claisse, 1976). One possible explanation for this relationship is indicated by the work of Pollack and Rifkin (1975) who observed that the sheaths of actin cables associated with anchorage-dependent growth were lost in cells grown in semi-solid medium, and that that this loss could be achieved by treatment with plasmin, the active proteolytic enzyme produced by plasminogen activation. Such results are consistent with our postulate that fibrinolytic activity may precede and be necessary for the induction of growth in agar in ENU-induced transformation of rat brain cells. Experiments are in progress, in this laboratory, to test this proposition.

It has been shown that plasminogen-activator production is associated with the invasive phase of the mouse trophoblast and possible migration of the parietal endoderm (Strickland *et al.*, 1976), and its involvement in tumour invasiveness has often been proposed. The finding that cells of an ENU-induced glioma are invasive *in situ* (Lantos, 1972) and that the re-injection of cultured glioma cells (high fibrinolytic activity) leads to the penetration of muscle and bone during tumour formation, is not inconsistent with this proposition (Lantos *et al.*, 1976). The invasiveness of tumourigenic E6 cultures when re-injected has also been noted (Claisse *et al.*, in preparation).

We wish to thank Mr D. P. Winslow for expert technical assistance. This investigation was supported by a grant from the Cancer Research Campaign to J.P.R.

### REFERENCES

ALKJAERSIG, N., FLETCHER, A. P. & SHERRY, S. (1959) ε-aminocaproic Acid: an Inhibitor of Plasminogen Activator. *J. biol. Chem.*, **234**, 832.

ÅSTEDT, B. & HOLMBERG, L. (1976) Immunological Identity of Urokinase and Ovarian Carcinoma Plasminogen Activator Released in Tissue Culture. *Nature, Lond.*, **261**, 595.

BERNIK, M. B. & KWAAN, H. C. (1969) Plasminogen Activator Activity in Cultures from Human Tissues. An Immunological and Histochemical Study. *J. clin. Invest.*, **48**, 1740.

CHRISTMAN, J. K. & ACS, G. (1974) Purification and Characterization of a Cellular Plasminogen Activator Associated with Oncogenic Transformation: The Plasminogen Activator from SV40-transformed Hamster Cells. *Biochim. biophys. Acta*, **340**, 339.

CLAISSE, P. J. & ROSCOE, J. P. (1976) The Inducibility of Glycerol Phosphate Dehydrogenase in Two Rat Glial Clones. *Brain Res.*, **109**, 423.

DRUCKREY, H., IVANKOVIC, S. & PREUSSMANN, R. (1966) Teratogenic and Carcinogenic Effects in the Offspring after a Single Injection of Ethylnitrosourea to Pregnant Rats. *Nature, Lond.*, **210**, 1378.

HINCE, T. A. & ROSCOE, J. P. (1977) Fibrinolytic Activity Associated with Rat Brain Cells Exposed Transplacentally to the Carcinogen Ethylnitrosourea. (Abstract). *Br. J. Cancer*, **36**, 401.

JONES, P., BENEDICT, W., STRICKLAND, S. & REICH, E. (1975) Fibrin Overlay Methods for the Detection of Single Transformed Cells and Colonies of Transformed Cells. *Cell*, **5**, 323.

JONES, P., LAUG, W. E., GARDNER, A., NYE, C., FINK, L. M. & BENEDICT, W F. (1976) *In vitro* Correlates of Transformation in C3H/10T½ Clone 8 Mouse Cells. *Cancer Res.*, **36**, 2863.

LANTOS, P. L. (1972) The Fine Structure of Periventricular Pleomorphic Gliomas Induced Transplacentally by *N*-ethyl-*N*-nitrosurea in BD-IX Rats. *J. neurol. Sci.*, **17**, 443.

LANTOS, P. L., ROSCOE, J. P. & SKIDMORE, C. J. (1976) Studies of the Morphology and Tumorigenicity of Experimental Brain Tumours in Tissue Culture. *Br. J. exp. Path.*, **57**, 95.

LAUG, W. E., JONES, P. A. & BENEDICT, W. F. (1975) Relationship between Fibrinolysis of Cultured Cells and Malignancy. *J. natn. Cancer Inst.*, **54**, 173.

LEAVITT, J. C., CRAWFORD, B. D., BARRETT, J. C. & TS'O, P. O. P. (1977) Regulation of Requirements for Anchorage-independent Growth of Syrian Hamster Fibroblasts by Somatic Mutation. *Nature, Lond.*, **269**, 63.

NAGY, B., BAN, J. & BRDAR, B. (1977) Fibrinolysis Associated with Human Neoplasia: Production of Plasminogen Activator by Human Tumours. *Int. J. Cancer*, **19**, 614.

OSSOWSKI, L., UNKELESS, J. C., TOBIA, A., QUIGLEY, J. P., RIFKIN, D. B. & REICH, E. (1973) An Enzymatic Function Associated with Transformation of Fibroblasts by Oncogenic Viruses. II. Mammalian Fibroblast Cultures Transformed by DNA and RNA Tumor Viruses. *J. exp. Med.*, **137**, 112.

PEARLSTEIN, E., HYNES, R. O., FRANKS, L. M. & HEMMINGS, V. J. (1976) Surface Proteins and Fibrinolytic Activity of Cultured Mammalian Cells. *Cancer Res.*, **36**, 1475.

POLLACK, R., RISSER, R., CONLON, S. & RIFKIN, D. B. (1974) Plasminogen Activator Production Accompanies Loss of Anchorage Regulation in Transformation of Primary Rat Embryo Cells by Simian Virus 40. *Proc. natn. Acad. Sci., U.S.A.*, **71**, 4792.

POLLACK, R. & RIFKIN, D. B. (1975) Actin-containing Cables Within Anchorage-dependent Rat Embryo Cells are Dissociated by Plasmin and Trypsin. *Cell*, **6**, 496.

POLLACK, R., RISSER, R., CONLON, S., FREEDMAN, V., SHIN, S.-I. & RIFKIN, D. B. (1975) Production of Plasminogen Activator and Colonial Growth in Semi-solid Medium are *In vitro*. Correlates of Tumorigenicity in the Immune-Deficient Nude Mouse. In *Proteases and Biological Control*, Ed. E. Reich, D. B. Rifkin & E. Shaw. N.Y.: Cold Spring Harbor Press, p. 885.

QUIGLEY, J. P., OSSOWSKI, L. & REICH, E. (1974) Plasminogen, the Serum Proenzyme Activated by Factors from Cells Transformed by Oncogenic Viruses. *J. biol. Chem.*, **249**, 4306.

RIFKIN, D. B. & POLLACK, R. (1977) Production of Plasminogen Activator by Established Cell Lines of Mouse Origin. *J. Cell Biol.*, **73**, 47.

ROSCOE, J. P. & CLAISSE, P. J. (1976) A Sequential *In vivo*—*In vitro* Study of Carcinogenesis Induced in the Rat Brain by Ethylnitrosourea. *Nature, Lond.*, **262**, 314.

ROSCOE, J. P. & CLAISSE, P. J. (1977) An Investigation of Ethylnitrosourea induced Carcinogenesis in the Rat Brain by an *In vivo*—*In vitro* Method. (Abstract). *Br. J. Cancer*, **36**, 401.

SCHULTZ, D. R., WU, M.-C. & YUNIS, A. A. (1975) Immunologic Relationship among Fibrinolysins Secreted by Cultured Mammalian Tumor Cells. *Expl. Cell Res.*, **96**, 47.

STRICKLAND, S., REICH, E. & SHERMAN, M. I. (1976) Plasminogen Activator in Early Embryogenesis: Enzyme Production by Trophoblast and Parietal Endoderm. *Cell*, **9**, 231.

UNKELESS, J. C., TOBIA, A., OSSOWSKI, L., QUIGLEY, J. P., RIFKIN, D. B. & REICH, E. (1973) An Enzymatic Function Associated with Transformation of Fibroblasts of Oncogenic Viruses. I. Chick Embryo Fibroblast Cultures Transformed by Avian RNA Tumor Viruses. *J. exp. Med.*, **137**, 85.

UNKELESS, J., DANØ, K., KELLERMAN, G. M. & REICH, E. (1974) Fibrinolysis Associated with Oncogenic Transformation. Partial Purification and Characterization of the Cell Factor, a Plasminogen Activator. *J. biol. Chem.*, **249**, 4295.

WECHSLER, W., KLEIHUES, P., MATSUMOTO, S., ZÜLCH, K. L., IVANKOVIC, S., PREUSSMANN, R. & DRUCKREY, H. (1969) Pathology of Experimental Neurogenic Tumors Chemically Induced During Prenatal and Postnatal Life. *Ann. N.Y. Acad. Sci.*, **159**, 360.

WU, M.-C., ARIMURA, G. K. & YUNIS, A. A. (1977) Purification and Characterization of a Plasminogen Activator Secreted by Cultured Human Pancreatic Carcinoma Cells. *Biochemistry*, **16**, 1908.

WU, M.-C., SCHULTZ, D. R., ARIMURA, G. K., GROSS, M. A. & YUNIS, A. A. (1975) Characteristics of Fibrinolysin Secreted by Cultured Rat Breast Carcinoma Cells. *Expl. cell Res.*, **96**, 37.


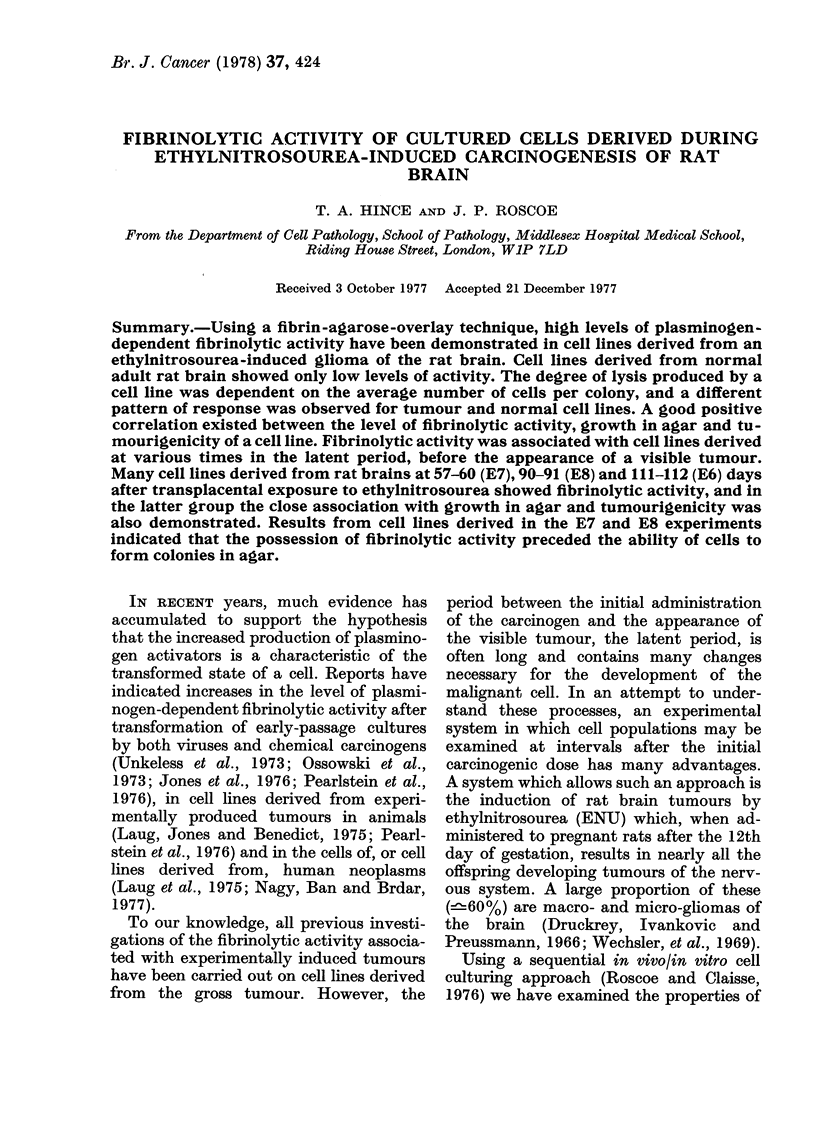

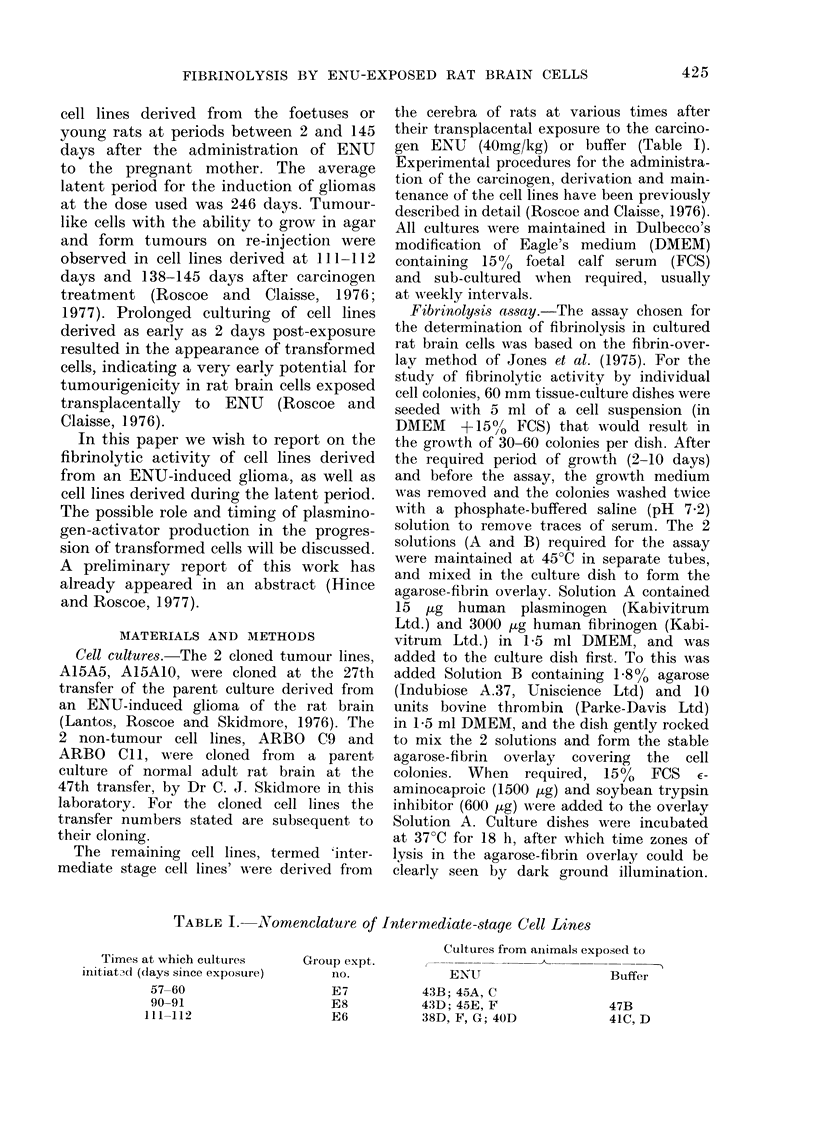

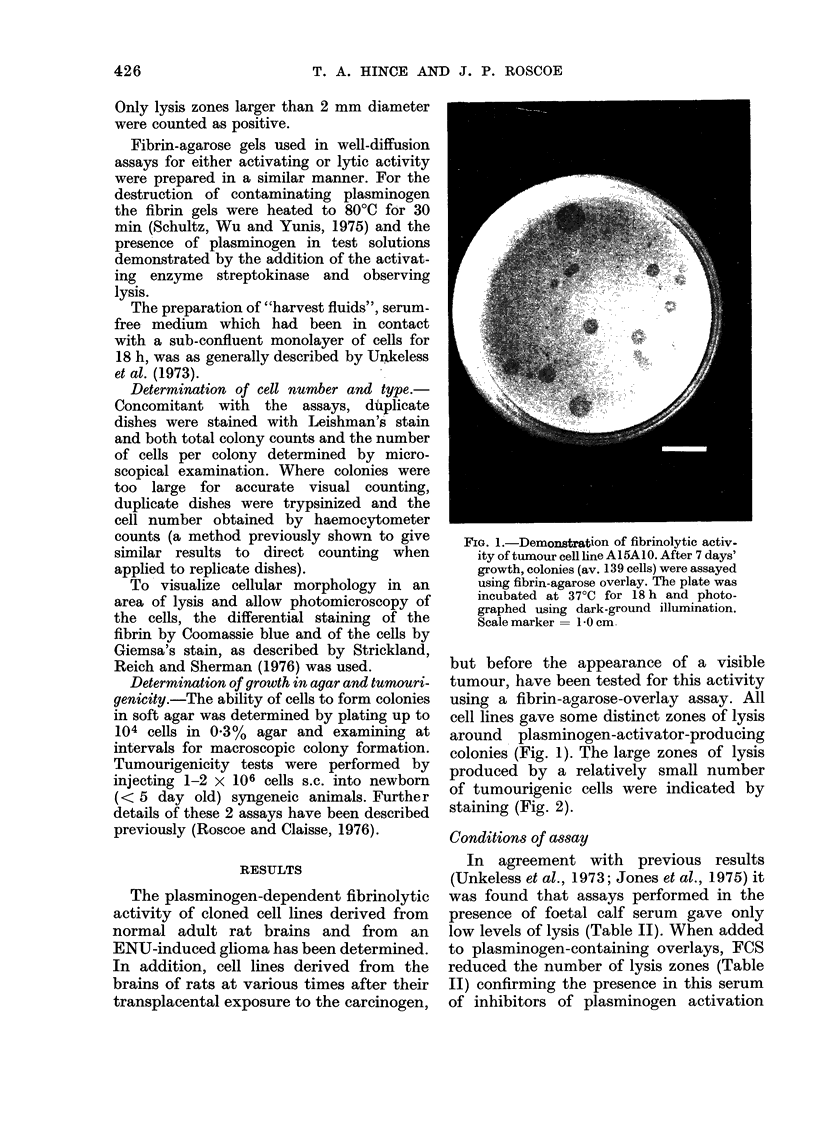

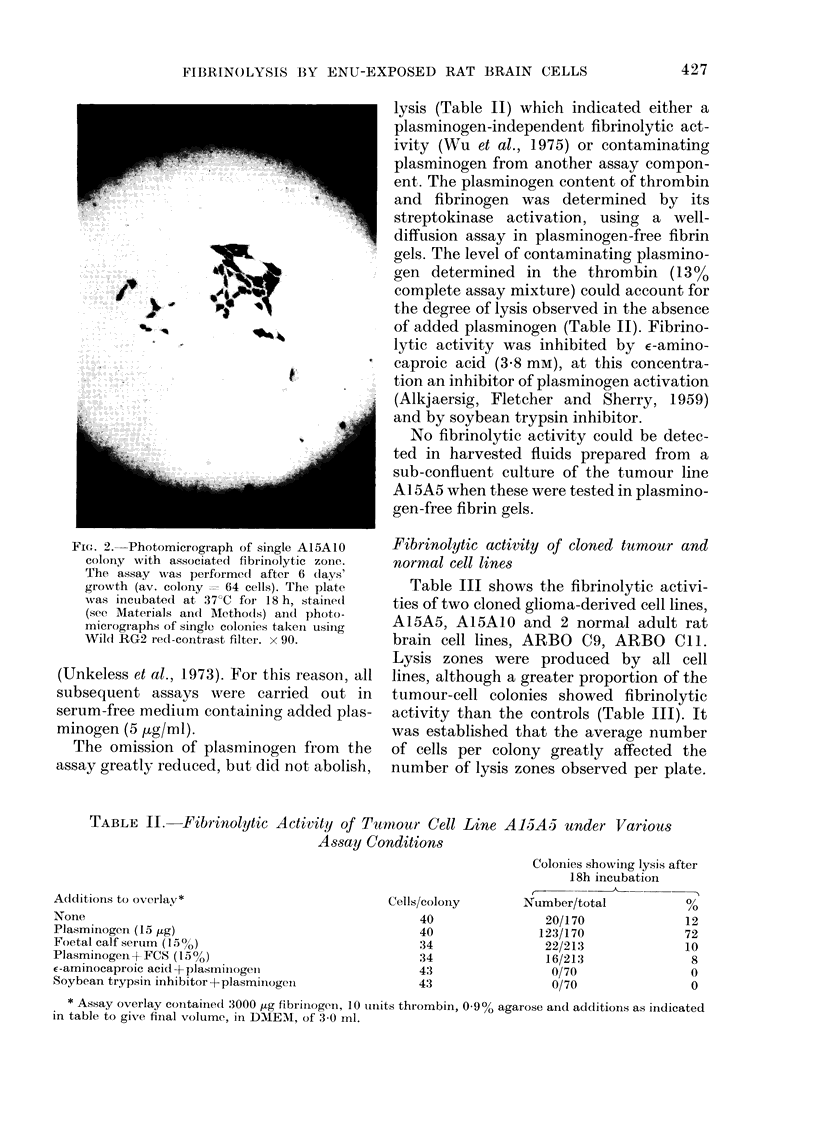

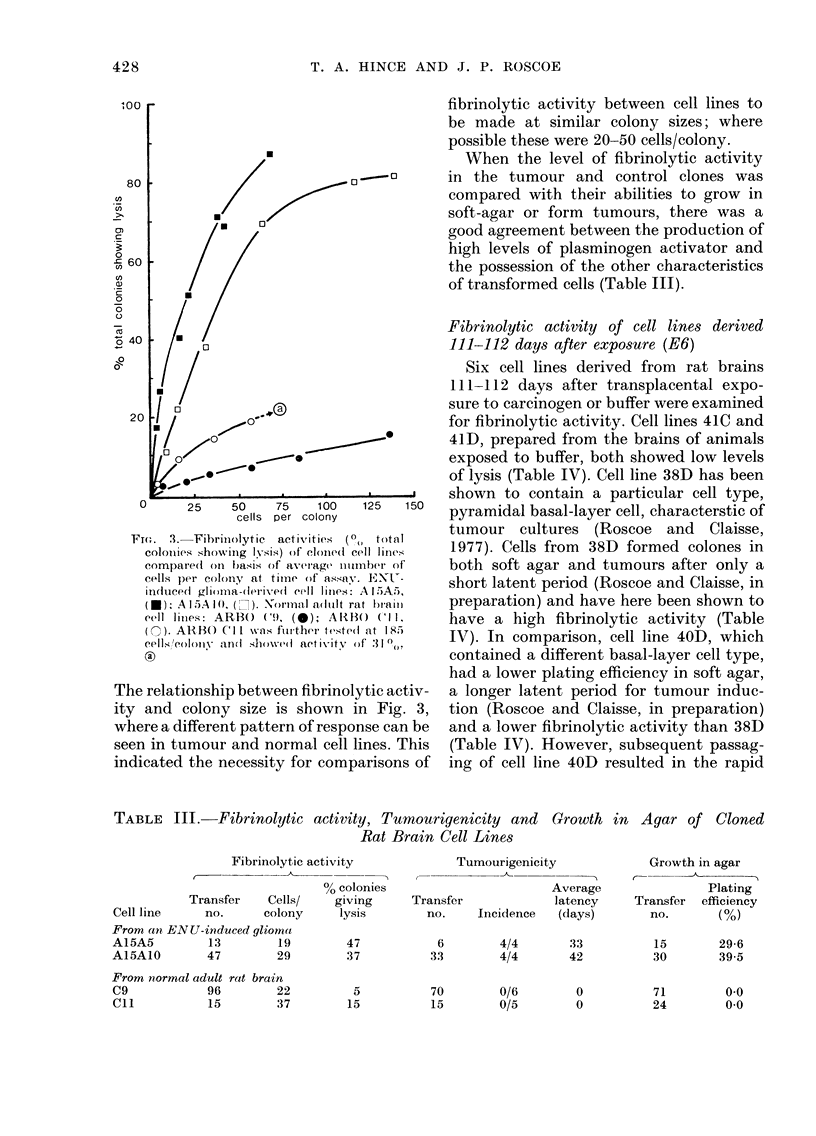

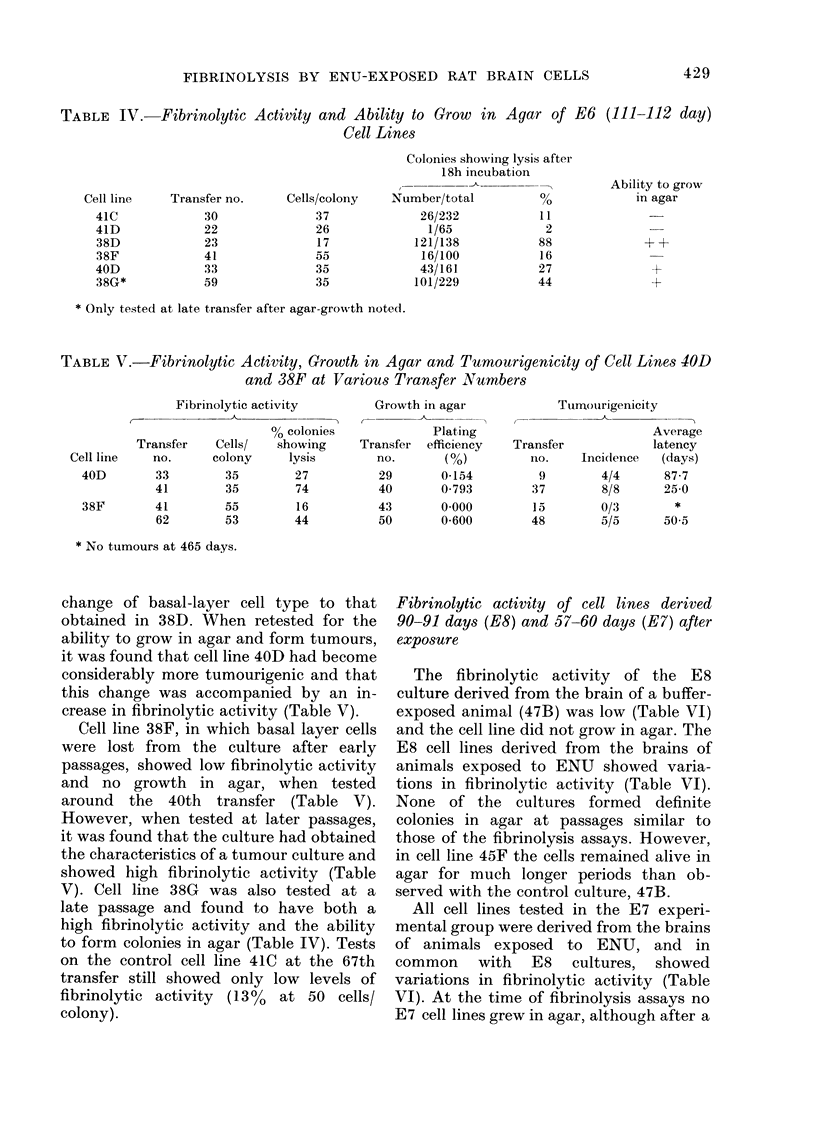

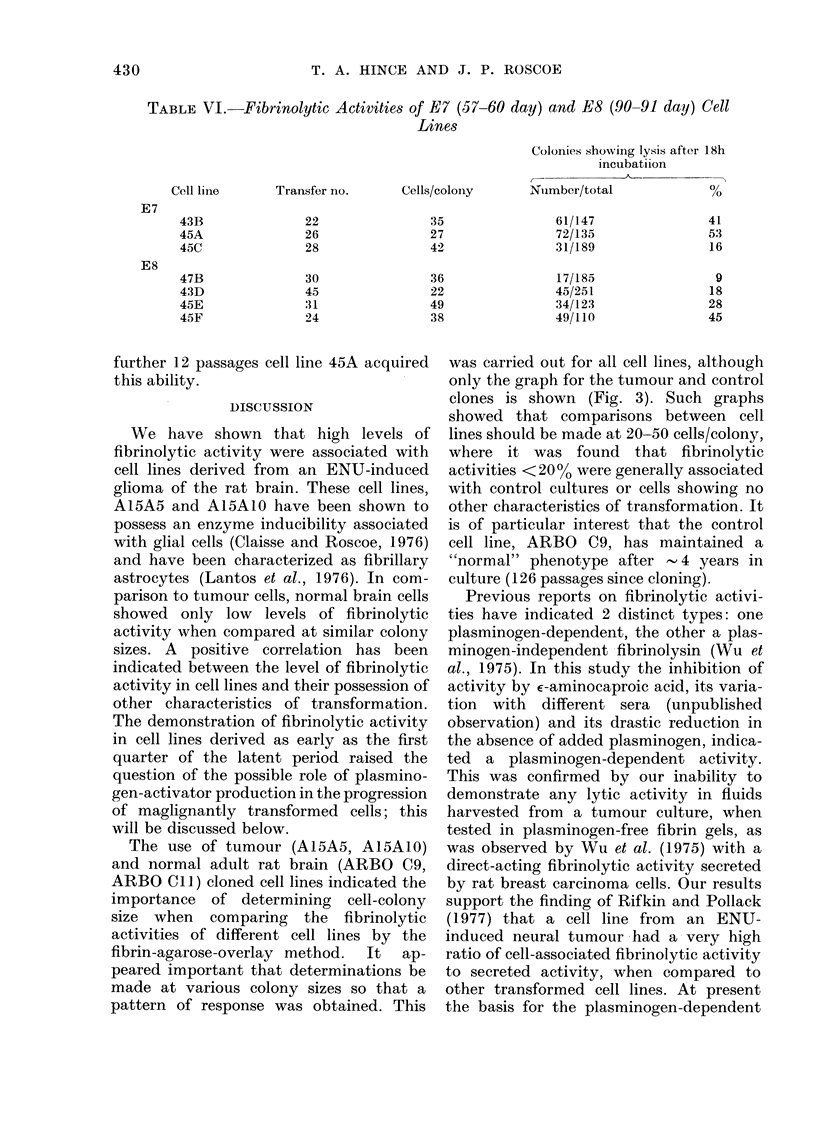

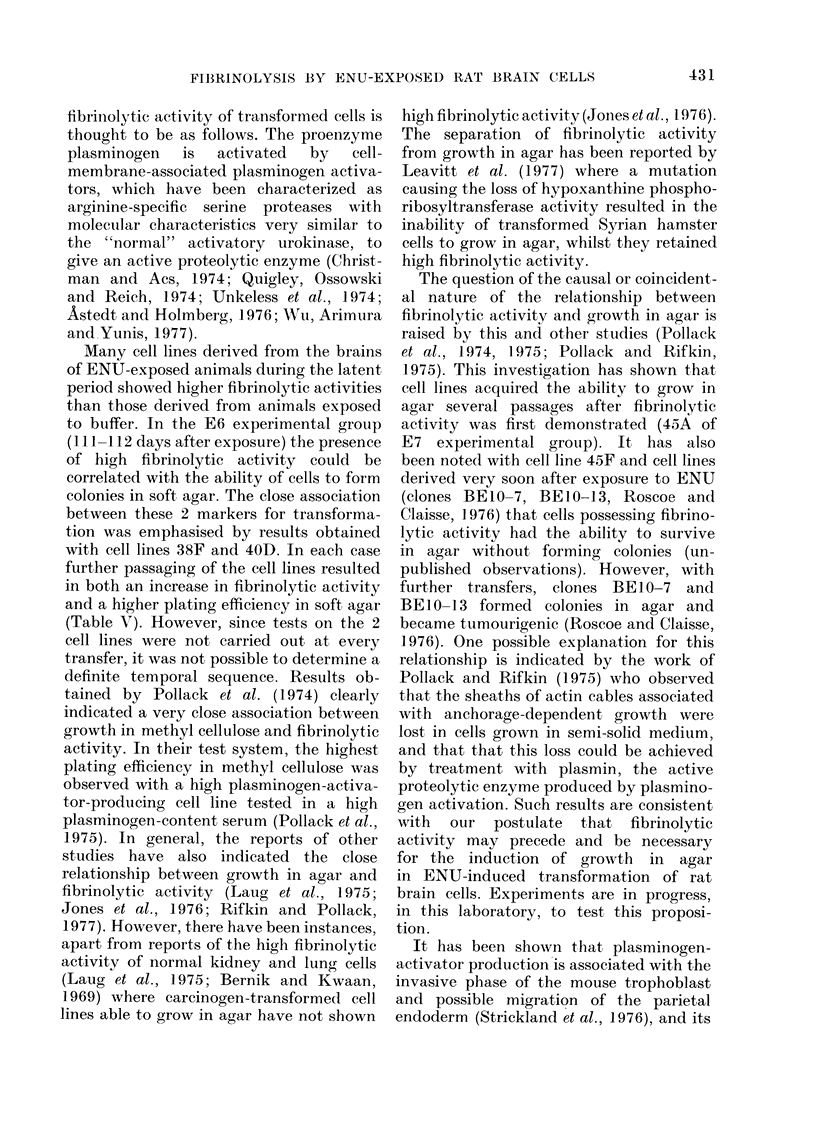

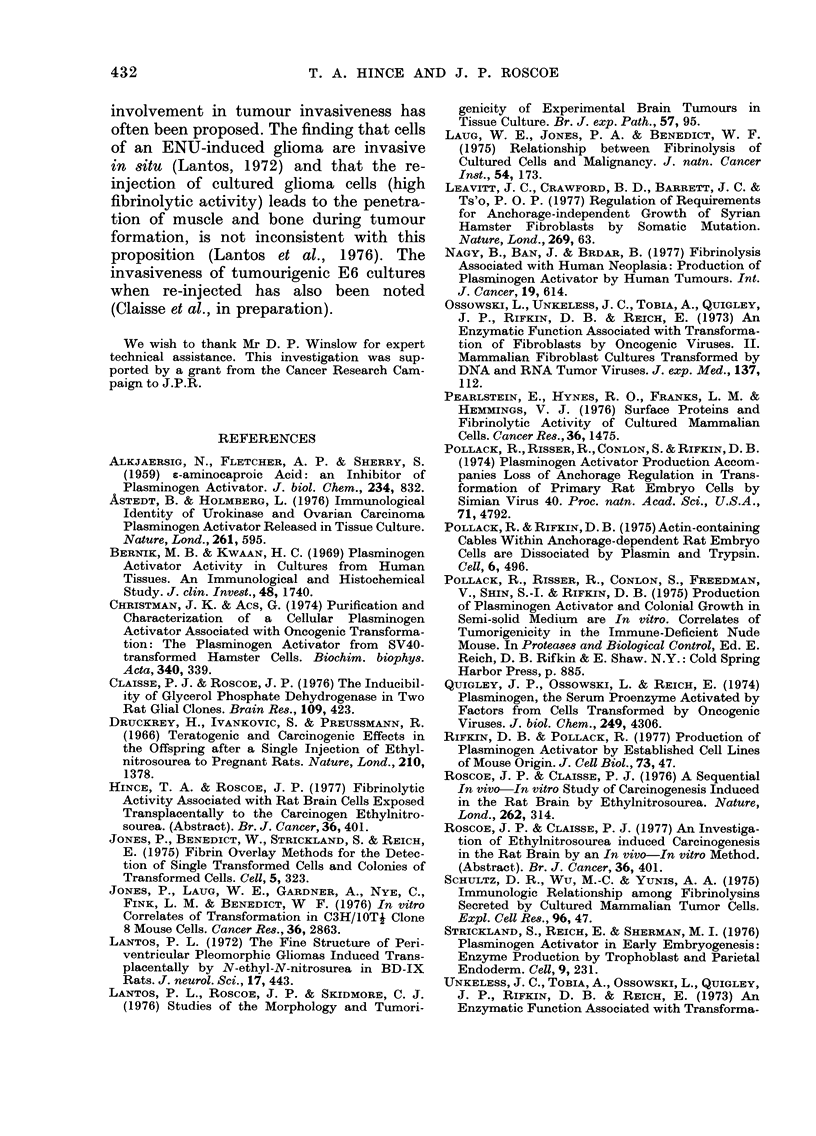

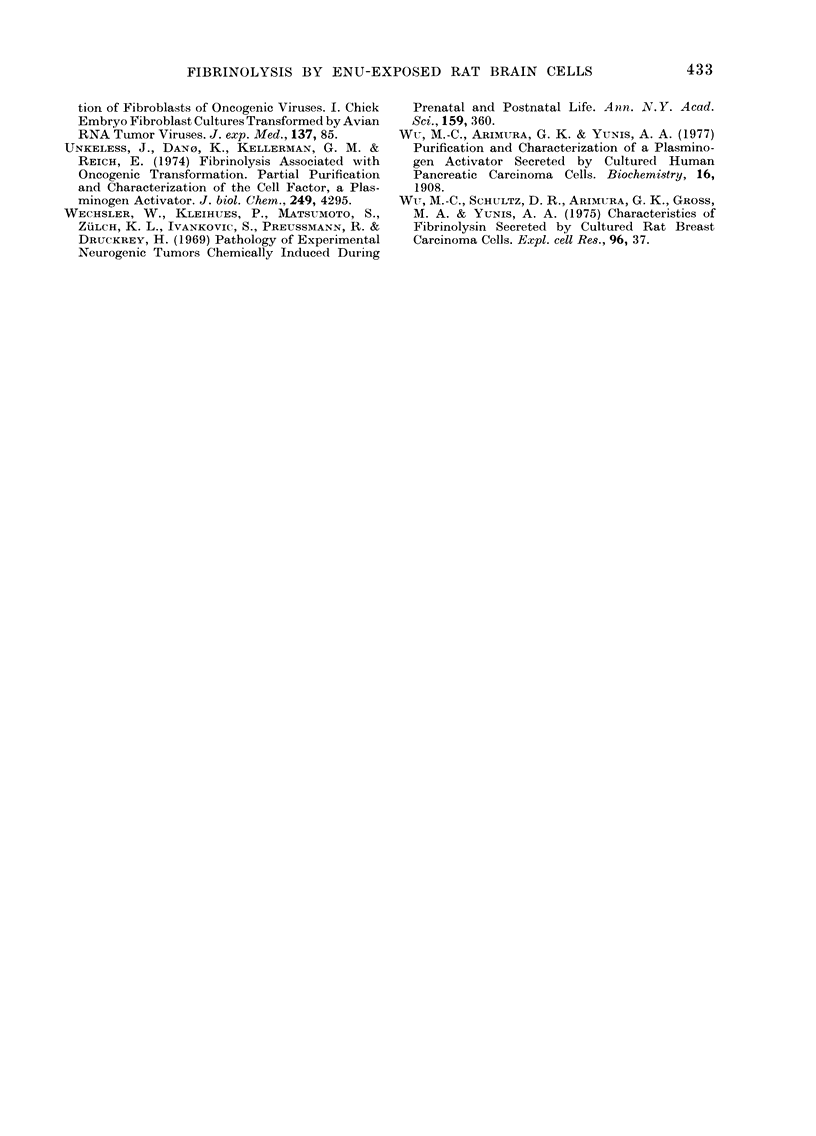


## References

[OCR_01007] ALKJAERSIG N., FLETCHER A. P., SHERRY S. (1959). xi-Aminocaproic acid: an inhibitor of plasminogen activation.. J Biol Chem.

[OCR_01011] Astedt B., Holmberg L. (1976). Immunological identity of urokinase and ovarian carcinoma plasminogen activator released in tissue culture.. Nature.

[OCR_01017] Bernik M. B., Kwaan H. C. (1969). Plasminogen activator activity in cultures from human tissues. An immunological and histochemical study.. J Clin Invest.

[OCR_01023] Christman J. K., Acs G. (1974). Purification and characterization of a cellular fibrinolytic factor associated with oncogenic transformation: the plasminogen activator from SV-40-transformed hamster cells.. Biochim Biophys Acta.

[OCR_01031] Claisse P. J., Roscoe J. P. (1976). The inducibility of glycerol phosphate dehydrogenase in two rat glial clones.. Brain Res.

[OCR_01036] Druckrey H., Ivanković S., Preussmann R. (1966). Teratogenic and carcinogenic effects in the offspring after single injection of ethylnitrosourea to pregnant rats.. Nature.

[OCR_01055] Jones P. A., Laug W. E., Gardner A., Nye C. A., Fink L. M., Benedict W. F. (1976). In vitro correlates of transformation in C3H/10T1/2 clone 8 mouse cells.. Cancer Res.

[OCR_01049] Jones P., Benedict W., Strickland S., Reich E. (1975). Fibrin overlay methods for the detection of single transformed cells and colonies of transformed cells.. Cell.

[OCR_01067] Lantos P. L., Roscoe J. P., Skidmore C. J. (1976). Studies of the morphology and tumorigenicity of experimental brain tumours in tissue culture.. Br J Exp Pathol.

[OCR_01061] Lantos P. L. (1972). The fine structure of periventricular pleomorphic gliomas induced transplacentally by N-ethyl-N-nitrosourea in BD-IX rats. With a note on their origin.. J Neurol Sci.

[OCR_01074] Laug W. E., Jones P. A., Benedict W. F. (1975). Relationship between fibrinolysis of cultured cells and malignancy.. J Natl Cancer Inst.

[OCR_01087] Nagy B., Ban J., Brdar B. (1977). Fibrinolysis associated with human neoplasia: production of plasminogen activator by human tumours.. Int J Cancer.

[OCR_01093] Ossowski L., Unkeless J. C., Tobia A., Quigley J. P., Rifkin D. B., Reich E. (1973). An enzymatic function associated with transformation of fibroblasts by oncogenic viruses. II. Mammalian fibroblast cultures transformed by DNA and RNA tumor viruses.. J Exp Med.

[OCR_01102] Pearlstein E., Hynes R. O., Franks L. M., Hemmings V. J. (1976). Surface proteins and fibrinolytic activity of cultured mammalian cells.. Cancer Res.

[OCR_01108] Pollack R., Risser R., Conlon S., Rifkin D. (1974). Plasminogen activator production accompanies loss of anchorage regulation in transformation of primary rat embryo cells by simian virus 40.. Proc Natl Acad Sci U S A.

[OCR_01132] Quigley J. P., Ossowski L., Reich E. (1974). Plasminogen, the serum proenzyme activated by factors from cells transformed by oncogenic viruses.. J Biol Chem.

[OCR_01138] Rifkin D. B., Pollack R. (1977). Production of plasminogen activator by established cell lines of mouse origin.. J Cell Biol.

[OCR_01143] Roscoe J. P., Claisse P. J. (1976). A sequential in vivo-in vitro study of carcinogenesis induced in the rat brain by ethylnitrosourea.. Nature.

[OCR_01155] Schultz D. R., Wu M. C., Yunis A. A. (1975). Immunologic relationship among fibrinolysins secreted by cultured mammalian tumor cells.. Exp Cell Res.

[OCR_01161] Strickland S., Reich E., Sherman M. I. (1976). Plasminogen activator in early embryogenesis: enzyme production by trophoblast and parietal endoderm.. Cell.

[OCR_01178] Unkeless J., Dano K., Kellerman G. M., Reich E. (1974). Fibrinolysis associated with oncogenic transformation. Partial purification and characterization of the cell factor, a plasminogen activator.. J Biol Chem.

[OCR_01194] Wu M., Arimura G. K., Yunis A. A. (1977). Purification and characterization of a plasminogen activator secreted by cultured human pancreatic carcinoma cells.. Biochemistry.

